# Navigating the Ethical and Methodological Dimensions of a Farm Safety Photovoice Project

**DOI:** 10.1007/s11673-023-10261-8

**Published:** 2023-05-23

**Authors:** Florence A. Becot, Shoshanah M. Inwood, Elizabeth A. Buchanan

**Affiliations:** 1 National Farm Medicine Center, Marshfield Clinic Research Institute, 1000 N Oak Ave, ML-1, Marshfield, WI 54449 USA; 2 School of Environment and Natural Resources, The Ohio State University, 132 Williams Hall, 1680 Madison Avenue, Wooster, OH 44691 USA; 3 Marshfield Clinic Research Institute, 1000 N Oak Ave, 1R3, Marshfield, WI 54449 USA

**Keywords:** Agricultural health and safety, Children, Farm women, Farm safety, Participatory research methods, Photovoice, Research ethics committees, Social, psychological, and privacy risks

## Abstract

Scholars have noted persistent high rates of agricultural health and safety incidents and the need to develop more effective interventions. Participatory research provides an avenue to broaden the prevailing research paradigms and approaches by allowing those most impacted to illuminate and work to solve those aspects of their lives. One such approach is photovoice, an emancipatory visual narrative approach. Yet, despite its broad appeal, photovoice can be hard to implement. In this article, we leverage our experience using photovoice for a farm children safety project to describe and reflect on the ethical and methodological aspects broadly relevant to agricultural health and safety topics. We first contextualize the tensions of navigating between photovoice, the research ethics committees (RECs) regulatory frameworks, and competing views on visual representations in agriculture. We then discuss the sources of risks to participants and researchers, how we addressed these risks, and how these risks unfolded during the research phase of the photovoice activity. We conclude with three lessons we (re)learned: the importance of collaborating with RECs, the need to increase preparation to limit psychological risks to participants and researchers, and avenues to augment the emancipatory power of photovoice in a virtual environment.

## Introduction

Internationally, farm children suffer from high rates of injuries and fatalities (Committee on Injury and Poison Prevention and Committee on Community Health Services 2001; International Labour Organization n.d.). In the United States, the country where these authors do most of our work, about thirty-three children are seriously injured in agricultural-related incidents every day and one child dies about every three days (National Children’s Center for Rural and Agricultural Health and Safety 2020). Such high risk exposure largely stems from the overlap of farm children’s home with their parents’ dangerous worksite (Morrongiello et al. 2008; Elliot et al. 2018), along with limited regulations on children’s presence on agriculture worksites in many countries (Radfar et al. 2018; Edmonds and Theoharides 2021; International Labour Organization n.d.). For over thirty years, one key recommendation for limiting children’s risk exposure has been supervision on a dedicated, off-farm site using paid or unpaid childcare. Despite this long-standing recommendation, progress has been insufficient (Gallagher 2012; Voaklander et al. 2019).

The agricultural health and safety literature has largely sought to understand the reasons why farm parents are not adopting these farm safety practices through a focus on their farm safety knowledge and behaviours (Lee, Jenkins, and Westaby 1997; Pickett, Marlenga, and Berg 2003; Westaby and Lee 2003) as well as their social and cultural norms (Neufeld, Wright, and Gaut 2002; Zepeda and Kim 2006; Elliot et al. 2018; Shortall, McKee, and Sutherland 2019). The context in which farm parents make farm safety decisions and the ways in which farm parents make sense of their decisions have received much less attention (Gallagher 2012; Lee et al. 2017; Elliot et al. 2018). Furthermore, we have yet to understand the extent to which farm parents are able and/or willing to use childcare to keep their children safe despite evidence from a range of countries that childcare is costly and/or unavailable (Ogbimi 1992; Shortall et al. 2017; Inwood and Stengel 2020). In other words, the limited effectiveness of the prevailing farm safety interventions documented in evaluation research (Gallagher 2012) could in part stem from inadequate understanding and incorporation of farm parents’ lived realities and how they consider their children’s safety. The limited effectiveness of prevailing interventions and insufficient progress in reducing agricultural injuries is not limited to farm children as scholars have noted similar challenges regarding farm adults (Rautiainen et al. 2008; Coman et al. 2020; Driscoll et al. 2022).

To broaden the research paradigms underpinning the farm safety research and the approaches used to develop recommendations, we leverage our experience with the “Women Raising Children on Farms” photovoice project. In particular, we describe and reflect on the ethical and methodological aspects to consider when developing and deploying a photovoice project for an agricultural health and safety project, with a focus on the research phase.^1^ Photovoice is a participatory and emancipatory visual narrative approach wherein participants themselves both illuminate and work to solve those aspects of their lives and challenges generally ignored by society and the literature (Wang and Burris 1997; Sutton-Brown 2014). As such, photovoice provides opportunities to broaden the agricultural health and safety field by asking farm parents to tell us about their realities, giving these parents a space to interact and reflect with parents in similar situations, and involving them as active developers of recommendations both realistic and acceptable to them. Scholars and community-based organizations have used photovoice as a research and empowerment tool with a range of underserved and under-represented populations. However, we are only aware of three photovoice projects in the English-language scientific literature on agricultural health and safety topics (De Castro, Krenz, and Neitzel 2014; Schwartz et al. 2015; Mott, Keller, and Funkenbusch 2017), one of which included children, a protected group in research ethics committee (REC)^2^ regulatory frameworks. None provided an in-depth description of the planning and deployment of the methods. The need to reconcile competing tensions between the grounding of photovoice in emancipatory social theories with the RECs regulatory frameworks grounded in the dominant biomedical research model can be particularly challenging for researchers working under REC jurisdiction (Brown et al. 2010; Anderson et al. 2012). Furthermore, a photovoice project on an agricultural health and safety topic contains an additional layer of complexity due to competing views on visual representations in agriculture, which we will discuss further below.

Our article contributes to photovoice’s rich literature, applying it to a topic for which it has seldom been used before. Acknowledging that the richness of the theoretical debates in the photovoice literature contrasts with the largely applied and positivist literature of agricultural health and safety, our article contributes by summarizing key tensions agricultural health and safety scholars wanting to use photovoice would face regarding RECs and norms around visual representations in agriculture. To be clear, there already exists extensive practical guidance including step-by-step planning of a photovoice activity, debriefing picture taking and activities, ensuring participants’ privacy and physical safety, and ensuring photo rights (Wang and Redwood-Jones 2001; Amos et al. 2012; Cox et al. 2014; Jongeling et al. 2016; Aboulkacem, Aboulkacem, and Haas 2021; Evans-Agnew, Rosemberg, and Boutain 2022). Still, scholars have noted that practical guidance to help researchers develop a photovoice project, particularly to support researchers’ adherence to the principles of both photovoice and RECs, remains limited (Yassi et al. 2016; Lenette et al. 2018; Teti 2019). Furthermore, every photovoice project generates a unique set of situational ethical and methodological dilemmas for both the participants and those initiating the activity (Lenette et al. 2018; McDonald and Capous-Desyllas 2021). We hope that our description of how we reconciled the multiple potential risks in our protocol for the research phase can provide a guiding framework to help other scholars think through their own projects.

## What is Photovoice?

In photovoice, participants engage in the role of researchers and knowledge creators by taking pictures and debriefing about their pictures. Participants then also often take on the role of educators and advocates by curating a photography exhibit targeted to their communities and decision-makers, calling attention to their realities and asking for solutions (Wang and Burris 1997; Sutton-Brown 2014). With epistemological grounding in feminism’s and Freire’s critical consciousness theories, photovoice invites participants into a reflection of their social, economic, and political realities (Wang and Burris 1997).

Despite its broad appeal among academics and community-based organizations, logistics and navigating risk to participants can make photovoice challenging to implement. In turn these limitations can limit the emancipatory nature of photovoice. From a logistical standpoint, a photovoice activity generally involves three phases: planning, deployment, and public engagement. In planning, team organizers consider who should participate, the logistics of group meetings, the activity’s focus, the cameras to be used, and picture management. Deployment includes recruitment of participants, training of participants (in technical and ethical aspects of picture taking), discussion of picture prompts (sometimes generated by the organizing team, other times in collaboration with participants), time for participants to take pictures, organization of the pictures to be shown during the group debrief(s), and group debrief(s) to facilitate group discussions around the pictures. Lastly, the public engagement phase often takes the form of a public picture exhibit. Not all projects include this last phase, which requires discussions with participants regarding picture displays, messaging, deciding who to invite, finding a public space and/or creating a website, assembling the pictures, sending invitations, and tending the exhibit (Wang and Redwood-Jones 2001; Cox et al. 2014; Jongeling, Bakker et al. 2016; Humpage et al. 2019). Unique considerations associated with picture taking and sharing must be made: organizers of a photovoice project need to consider, among many things, the safety of participants when taking pictures, the consent process to take pictures of other people, picture rights and ownership, and negative judgements made about participants and/or their community. Risk mitigation is central to RECs, and so we turn next to the tensions between the participatory nature of photovoice and REC regulatory frameworks.

## Tensions Between REC Regulatory Framework and Photovoice

When planning and deploying participatory research methods such as photovoice, scholars must navigate an REC framework largely drawn from the biomedical research model (Brown et al. 2010; Anderson et al. 2012). In what follows, we draw on the research ethics and photovoice bodies of literature to map three tensions most relevant to the agricultural health and safety field to think through in the planning and implementation of a photovoice activity. Broadly speaking, these tensions are in part connected to the concept of “ethic creep” which is connected to the bureaucratization and expanding reach of RECs (Haggerty 2004; Guta, Nixon and Wilson 2013)

The first tension stems from photovoice’s focus on marginalized populations, which may overlap with populations RECs consider vulnerable (Flicker et al. 2007). In the United States, these protected groups include children, prisoners, individuals with impaired decision-making capacity, and economically and educationally disadvantaged persons (45 CFR 46.107. (a)).^3^ In the context of agriculture, several of these REC-protected groups are also considered vulnerable by health and safety experts, including children and hired farm workers (whose immigration, economic, and educational positions may disadvantage them). While not explicitly considered a vulnerable population by the agricultural health and safety field, farmers experiencing financial difficulties could also be considered a protected group because of their economic disadvantage. Strong rationales exist for protecting these groups (Seidelman 1996; Corbie-Smith 1999). Still, scholars have noted both the paternalistic and overzealous nature of RECs’ treatment of vulnerable populations (Edwards, Kirchin and Huxtable 2004; Flicker et al. 2007; Miller and Wertheimer 2007; Rivera 2012; Cross, Pickering and Hickey 2015; Resnik 2015; Yanar et al. 2016; Humpage et al. 2019). These extra protections may potentially alienate, stifle, or silence voices of already-marginalized populations (Flicker et al. 2007; Boxall and Ralph 2009; Perry 2011; Ponic and Jategaonkar 2012; Yanar et al. 2016; Teti 2019; McCracken 2020). Therefore, planning the photovoice activity needs to include discussions around how strict protections may deter participants from meaningfully sharing their realities and perspectives.

The second source of tensions stems from changing norms around picture taking and sharing, the visual and public nature of photovoice, and how RECs approach privacy risks. RECs consider how data are collected, stored, disseminated, and destroyed. To minimize privacy risks, RECs emphasize anonymous and confidential data while considering data sensitivity and consequences should data be released or repurposed beyond the original research (Medical Ethics Advisor 2021). Yet the focus on privacy conflicts with photovoice in two ways. First, its visual nature means that participants may take pictures with identifying features of themselves and others (i.e., potential non-research participants). In turn, the inclusion of non-research participants raises questions about their consent, especially for sharing the picture with others (SACHRP 2022). Second, photovoice is by design a public endeavour wherein photo debriefs commonly occur among a group of research participants and the curated public photo exhibit is a foundational aspect (Wang and Burris 1997; Wang and Redwood-Jones 2001). As such, in both the planning and deployment of the photovoice activity, scholars must think through who can be included and which identifying features are sharing-appropriate. They also must consider how this consent process could impact participation and results (Hannes and Parylo 2014; Yanar et al. 2016; Humpage et al. 2019). For example, if an organizing team fears that the cumbersome consent process for non-research participants could deter participation, they may instead ask that participants do not take pictures of people and/or that these pictures may only be shared during the photovoice debrief (not during the public exhibit). Last, in thinking through the appropriate level of review to apply for and who can be included, researchers need to contend with the ways in which a focus on preserving anonymity could at the same time stifle and disempower participants (Yanar et al. 2016).

The third source of tension stems from how decisions in a participatory projects are made between researchers and participants and around who “owns” the project—in other words, how power is shared. Traditionally, an REC protocol requires the researcher to provide information on recruitment, sampling strategies, research instruments, consent process, and data analysis before the project can be implemented. Because researchers are generally not allowed to engage with participants until the protocol has been reviewed, this means all key decisions are made without input from research participants. This approach conflicts with participatory research approaches calling for the involvement of all in the making of decisions (Wang and Redwood-Jones 2001; Call-Cummings, Hauber-Özer, and Ross 2020; Evans-Agnew, Rosemberg, and Boutain 2022). For example, the implementation of a community advisory board is a well-recognized solution, so that the community or population involved in the study has representation during those planning stages. Furthermore, RECs consider researchers the “owner” of the project, as do the participants, usually (Catalani and Minkler 2010; Call-Cummings et al. 2019). No matter how much decision sharing happens between the researcher and participants, funding, institutional, and societal power structures reinforce this ownership. To realize the emancipatory nature of photovoice, some researchers call for rethinking and disruption of that power structure; Evans-Agnew, Rosemberg, and Boutain (2022) and Call-Cummings, Hauber-Özer, and Ross (2020) provide practical guidance on approaches for greater participant–researcher power sharing.

## Tensions Between Visual Representations in Agriculture and Photovoice

The ethics of visual representations have been a key focus of photovoice scholars (Wang and Redwood-Jones 2001; Teti et al. 2012; Hannes and Parylo 2014). The theoretical and practical discussions arise from the ethical imperative to “do no harm” to participants and their communities. Combined with legal freedom of expression and inquiry and privacy law (Wang and Redwood-Jones 2001), this ethical tenet informs decisions regarding where pictures can be taken, who can be in them, which consent is to be given for the pictures, what is adequate to show, who owns them, and how they can be used. Responses to these questions identify risks to participants and aid the development of REC protocol and the photo ethics training. The answers further inform how participants and their communities might perceive their own visual representations or how they manage picture subjects (Hannes and Parylo 2014). A photovoice project on an agricultural health and safety topic requires the navigation of competing views on visual representations in agriculture. These competing views are connected to the role of social media in agriculture, social norms in the agricultural health and safety field, and ag-gag laws (laws that hinder the recording of agricultural operations).

Easing such projects, farmers have adopted camera phone and social media similarly to the general population, indicating the farm population is likely comfortable with taking pictures and sharing. In other words, what is asked of participants during a photovoice project is likely not out of the ordinary. This normalization should revise exposure risks that concern RECs. With that said, the nascent literature on the use of social media in agriculture has found that the farm sector sometimes carefully crafts social media messages about farmers’ identity, family farm traditions, and good farm practices for positive light in public image and marketing (Canziani et al. 2020; Daigle and Heiss 2021; Riley and Robertson 2021; Castro and Pini 2022; Riley and Robertson 2022). Thus, the “positivity bias” farm populations share with other groups complicates sharing considerations.

Social norms in the agricultural health and safety field and ag-gag laws further complicate a photovoice project. Farm safety experts have long used visual narratives to communicate safety risks and strategies (see, for example, Telling the Story Project (2019); Cultivate Safety (n.d.)). However the field has implicitly and explicitly adopted a norm whereby farm safety experts avoid visually representing dangerous practices, out of fear that such representation could be construed as an endorsement.^4^ For projects focusing on farm children, showing a child in a dangerous situation (for example, a child on a tractor) could also be seen as evidence of child endangerment, even if in most countries, there are no laws preventing farm parents from bringing their children to the worksite (Miller 2012; Reid-Musson, Strauss, and Mechler 2022). Ag-gag laws, first created in the 1990s in the United States to limit the sharing of agricultural production information, particularly livestock, to the public, also influences depictions of the farm site (Ceryes and Heaney 2019; Whitfort 2019).^5^ Australia, Canada, and France have since adopted similar laws. Overall, these laws prohibit the capturing and dissemination of visual representation through pictures and videos, with negative consequences on free speech, whistle blower protection, and transparency in agriculture (Lacy 2013; Robbins et al. 2016). Ceryes and Heaney (2019) posit that ag-gag laws could have a chilling effect on occupational safety, health surveillance, and research, given the potential legal repercussions of releasing material that could indicate abuse or misconduct. Ag-gag laws raise questions about the extent to which a photovoice project focused on farm works could be construed as whistle blowing, while the increased climate of opacity around agricultural practices could deter farmers or farm workers from participating in a photovoice project. We now turn to a discussion of our photovoice project and our approach to navigating tensions connected to visual representation in agriculture and the REC regulatory framework.

## Background on the “Women Raising Children on Farms” Photovoice Project

The “Women Raising Children on Farms” photovoice project is part of a five-year mixed methods and multi-state research project funded by the U.S. National Institute of Occupational Safety and Health (National Children’s Center for Rural and Agricultural Health and Safety 2021). The overall goal of this five-year project is to understand links between childcare arrangements and farm children safety and to develop recommendations that would ease access to childcare. As part of the qualitative phase of our exploratory sequential design (Creswell and Plano Clark 2017), two rounds of photovoice activity succeeded a series of eleven focus groups with sixty-seven women raising children on farms in three U.S. states (Ohio, Vermont, and Wisconsin). These women were recruited through farm service providers and farm organizations who shared the recruitment information through their social media accounts and listservs. Our choice to include a photovoice activity in this project was driven by three main factors aligned with common uses of photovoice. First, the lived realities of raising children on farms have been invisible, and the work of caring for the children largely remains “women’s work,” a group traditionally underserved by existing resources (Shortall 1996; Barbercheck, Brasier, and Kiernan 2009; Becot, Inwood, and Rissing 2022). Second, we wanted to develop recommendations with women raising children to ensure such recommendations are acceptable and realistic. This departs from the prevalent expert-based model for the development of interventions in the agricultural health and safety field. Third, scholars who have conducted focus groups with farm women have noted they value hearing other women sharing similar realities and making connections, an experience these scholars indicate can raise critical consciousness and hold emancipatory potential for participants (Pini 2002; Trauger et al. 2008).

We conducted two rounds of the photovoice activity to account for seasonal variations in agricultural production, to capture differences in childcare arrangements across the school year, and to build trust with participants. Thirty-three women participated in the first round in March 2022 and nineteen participated in the second round in June 2022. For each of these rounds we provided three picture prompts and asked women to email us eight to ten pictures. These picture prompts were: 1) What do you normally do with the children during the day? 2) When children are on the farm with you, what do you do to keep them safe? 3) How does thinking about juggling children, farm work, taking care of the household, and off-farm work make you feel? 4) We often hear that it takes a village to raise children, what does your village look like? 5) When juggling the children and keeping them safe, farm work, taking care of the house, and off-farm work? 6) What makes your day easier? What makes your day harder? Group debriefs were conducted using a series of prompting questions grounded in the principles of the SHOWed technic (Wang and Redwood-Jones 2001): 1) Describe your picture and tell us what is happening. 2) Why did you take a picture of this? 3) What does this picture tell us about [prompts connected to the picture prompts]? 4) How does this picture reflect the childcare/schooling options that you do or do not have access to? 5) What do you want farm policy decisions-makers, farm organizations, and farm service providers who focus on farm safety and farm business to take away from your picture and do about it? After a participant had talked through their pictures, we invited reflections and questions from others. In line with the photovoice literature, a vast majority of the pictures generated rich discussions, and we only had time to discuss one to three pictures from each participant. At the end of the group debriefs, we asked participants their thoughts on where they would like the exhibit to be shown and who should see it. We received 377 pictures and the group debriefs generated twenty-eight hours of audio recordings. While in this article we focus on the methodological and ethical considerations of the research phase, we touch on implications for the photo exhibit when relevant. Overall, the women who participated were from farms of a variety of scale and type of commodities produced. Most were biological mothers, though a few were foster and step-mothers. All had at least one child under the age of eighteen.

## Development of the REC Protocol for “Women Raising Children on Farms”

Development of the REC protocol was a close collaboration between the project research team (Becot and Inwood), with previous experience developing one photovoice protocol, and an REC director (Buchanan) who is also a research ethicist. Over the course of three months, we had regular phone and email interactions to develop the project protocol and reconcile regulatory and representational challenges. In particular, our initial primary concern was the social norm of not showing dangerous practices, when we expected the project to yield just such visual representation. Furthermore, in conversations with agricultural health and safety colleagues, we were advised to provide farm safety recommendations to participants if we should see pictures of dangerous practices and/or should women discuss dangerous practices during the group debrief, a recommendation we felt would be antagonistic to photovoice’s grounding in Freire’s (2000) pedagogy of the oppressed.

Thinking holistically about the risks to participants that RECs seek to minimize, we identified three potential risks to participants (privacy, social, and psychological) and determined that the magnitude of these risks to participants were minimal for two main reasons.^6^ First, the topic of the photovoice activity (parenting on farms and keeping children safe) resembles topics commonly discussed among farm parents and should not be perceived as intrusive. Second, as discussed above, broad use of social media means taking and sharing pictures, including of children, is a daily occurrence for many, including for farmers.

As we now turn to a discussion of risk, we note that we elected to decouple the knowledge-generation phase (i.e., research phase) from the action-phase (i.e., outreach phase) for the purpose of the REC protocol. In what follows, we focus on describing the research phase by focusing on the privacy risks, social risks, and psychological risks to participants. While not discussed in the REC protocol, we also discuss social and economic risks to researchers. This is because we discussed these risks when planning the project and because these risks have the potential to limit the emancipatory nature of a photovoice project on an agricultural health and safety topic.

### Privacy Risks for Women and Their Families Due to the Visual and Public Nature of Photovoice

#### Source(s) of Risk

The main risks to privacy were associated with the visual and public nature of photovoice. Yet, despite privacy risks being common in photovoice and the extensive guidelines (for example, Wang and Redwood-Jones 2001; Bugos et al. 2014), they led to the most discussions among our group and generated the most comments from the REC reviewers. We needed to address which people participants could include in their pictures, if any; whether pictures could include people’s identifying features (e.g., faces, tattoos); and the type of permission (e.g., written, oral, none) participants would be required to obtain to include other people in their pictures. At stake were the burden of participation, the efficacy and accuracy of findings, and photovoice’s central empowering principle.

#### Addressing the Risk

Established group research practices for photovoice guided our approach to limiting privacy risks. Perhaps the most important decision was made early on when deciding to separate the outreach and research components, since most risks to privacy stem from the public photo exhibit (Groot, Schrijver, and Abma 2021). Besides simplifying the protocol, the decoupling addressed tensions between photovoice and the REC regulatory framework regarding power. The two rounds of photovoice activity should provide time for participants to feel comfortable with the activity, other participants, and research team. Time during the group debriefs was allocated for preliminary discussions to plan the photo exhibit: where it should take place, who should see it, and what should be communicated. The research team could then incorporate this information when developing the REC protocol for the outreach phase.

Mitigating research phase risks centred on two junctures: when the picture is taken and when it is shared during group debriefs. When taking the picture, participants were instructed about location and inclusion of people. The consent form and the photovoice activity training material informed participants they could take pictures from any location as long as they obtained verbal permission from the owner in private settings other than their own (e.g., a home, a farm, a childcare centre). Participants were instructed that people in pictures are optional but could include selfies or a picture of themselves taken by someone else and/or of people over the age of eighteen. Participants could take pictures of people under eighteen only if they were the children’s parent or legal guardian. Adults and children needed to be able to answer simple questions, such as “is it OK if I take your picture?” and “is it OK if I show your picture to people you do not know?” Individuals over eighteen and children able to understand simple questions were to verbally agree to have their picture taken and shared with researchers and other research participants. We provided ideas for preserving privacy such as taking pictures from the back or below the head, or blurring identifying features from the picture (which we offered to do for participants). While it is a common approach in photovoice to seek written consent and for sharing the picture in dissemination efforts (for example, Wang and Redwood-Jones 2001; Bugos et al. 2014), we justified the sufficiency of an oral consent for our low-risk activity, pointing to contemporary societal practices. Lastly, given the picture prompts, our participants would likely take pictures of people from their social networks such as family members and friends with whom they likely have established trusting relationships (Humpage et al. 2019).

Sharing pictures during the research phase would occur during the group debrief and dissemination of findings. The consent form and group debrief introduction instructed participants to not share the content of the group debriefs (i.e., pictures and discussions) beyond the bounds of the project. These instructions also advised them we could not guarantee other participants would not share outside the project. Another strategy to preserve privacy involved giving participants full control over their cameras and pictures shared. Participants had the option to use their digital camera or one from the project. They were asked to email their pictures to the researchers ahead of the group debrief. While none of the participants borrowed a project camera, they would have been instructed to delete the pictures before sending the camera back had they borrowed one. The consent form indicated that by sharing pictures with the research team, they gave permission for their pictures to be included in presentations and publications. This was an important aspect of obtaining consent to use material as commonly required by academic journals. However, to preserve their privacy, and aligning with the research community’s common practices, we would only use pictures that included no identifying features in presentations and publications about the research.

### How Risk Unfolded During the Photovoice Activity

A close look at the participants’ pictures provides insights towards participants’ concerns around their privacy. Out of the 256 pictures shared by thirty-three participants for the first group debrief, all participants sent pictures of their children and over two-thirds sent a picture of themselves. More than eight in ten pictures with people included identifying features while only two participants asked us to blur parts of their pictures (in one case, their own children; in another, people from outside their family unit). Based on the group debriefs, it appears that some participants included pictures of children such as nieces/nephews or other children in childcare settings. In other words, risk to privacy did not seem to concern participants, with some not fully following the inclusion guidelines. It was not uncommon for participants to explain that they frequently take pictures to remember, and some shared that they post personal pictures on social media, comments which underscore the rationale we used in our REC application.

### Social Risks from Showing Children in the Dangerous Farm Worksite

#### Source(s) of Risk

Social risks to participating women and their families were connected to REC classification of vulnerable groups and the nature of the pictures participants were asked to take. The project focuses on their caregivers’ duties, and as such their pictures might include children, a group considered vulnerable in U.S. regulations (45 CFR 46.401). Because parents frequently bring their children to the farm worksite, and because of the dangerous nature of that worksite, the likely depiction of dangerous situations in pictures would violate the social norms of the agricultural health and safety field. In turn the pictures could potentially provide evidence of child endangerment.

#### Addressing the Risk

Previous photovoice literature has addressed social risks of participants taking pictures connected to illegal or socially unacceptable activities such as drug use or prostitution, including by vulnerable groups (Capous-Desyllas and Forro 2014; Carlberg-Racich 2021). One of the key principles of photovoice is to protect participants and their community’s safety and social standing by avoiding taking and sharing of incriminating pictures of the participants, those they include in their pictures, and their broader communities. We provided picture ethics training following the presentation of the photovoice activity, explaining both the importance of only sharing pictures they are comfortable with others seeing and also how pictures of children at the worksite could be perceived. We reiterated this in the consent form describing risks and mitigation strategies. Participants were encouraged to ask a family member or friend for advice before sending it to the researcher if they were not sure about how the picture might be perceived.

Another strategy to limit participant’s risk was ensuring adequate researcher training to recognize danger signs. In addition to our own knowledge of common child-rearing practices in agriculture and farm safety recommendations, we consulted resources from our states’ child protection services for recognizing the signs of child endangerment. None of the researchers are considered child abuse mandatory reporters by their states of residence or by their institutions. We included this information in the consent form and shared the approach that we would take should concerns about child endangerment arise; we stated that if we saw any potentially adverse events we would first seek guidance from our RECs on how to proceed instead of first contacting the authorities. Our overall goal was to avoid escalating a potentially problematic situation too quickly while also protecting the researchers with a documented process of reporting should the need arise.

#### How Risk Unfolded During the Photovoice Activity

As expected, participants shared numerous pictures of their children on the worksite. Common scenes included children in baby strollers in the barn or strapped in a car seat on a tractor, children playing with toys in the barn, children doing chores such as feeding animals, and children standing near their parents working. Considering current safety guidelines, some of these pictures appear to depict children in a potentially dangerous situation. For example, farm safety experts have long advised that even if strapped in a car seat, young children should never be on machinery due to the sounds, vibrations, and potential to fall off the equipment. However, the picture captions and the photovoice debrief provided rich context to understand why the children were in the worksite and pointed to how women frequently engaged in risk-benefit analyses to lower their children’s exposure to risk. For example, one participant showed a picture of her child alone in an ATV with an enclosed cab. She explained that what we could not see on the picture was the heavy machinery moving around in the farm ground. For this participant, having her child in a vehicle for which she could not open the door was her way to minimize the risks. Reflecting on the pictures and captions that participants shared, we do not believe that these pictures diverged from the norm of what we have seen in agriculture. Furthermore, the accompanying captions and group debriefs illustrated the extent to which most participants astutely understood dangers on the farm yet were limited in their ability to adopt best practices due in part to limited resource access. This contrasts with prevalent farm safety interventions intended to address knowledge gaps (Gallagher 2012). During several group debriefs, participants reflected on each other’s pictures, commenting on similarities in their day-to-day and exchanging tips on how to juggle parental and professional duties. In sum, the photovoice activity gave participants a space to share their lived-realities, for creating reflection and knowledge with their peers.

### Psychological Risk from Women Reliving Their Daily Stresses

#### Source(s) of Risk

The psychological risk to women stems from reliving stresses associated with their triple burden of care work, farm work, and off-farm work. Such stress could be experienced by taking and debriefing about their pictures and their feelings. Literature documenting the heavy stress faced by farm parents, especially women, has assessed this risk (Berkowitz and Perkins 1984; Rissing, Inwood, and Stengel 2021). However, while the photovoice activity raises a psychological risk, it also has cathartic potential. Farm women have reported feeling isolated and invisible; the focus groups enabled them to meet other women with similar experiences and to form new connections (Pini 2002; Trauger et al. 2008).

#### Addressing the Risk

Minimal risk research requires researchers to diminish potential psychological discomforts to a level no greater than what participants ordinarily encounter during the performance of psychological tests. Preparatory steps included informing participants through the consent form about the potentially distressing nature of the project and providing adequate training for the researchers. While we as researchers have extensive qualitative data collection experience with farm populations, our experience conducting research on potentially distressing topics is limited. Given that, we had recently gone through the “Mental Health First Aid,” an online skill development to assist someone experiencing a mental health or substance use crisis (Mental Health First Aid USA 2020), we leveraged that training, along with other common REC strategies, to develop the following strategies. First, in the introduction of the group photovoice debrief, we indicated that participants could leave the debrief at any time and that they could use the Zoom private chat function to interact with researchers. Second, if we were to see signs that a participant was experiencing distress, we would privately message them and offer a phone debrief and share mental health resources. In these cases, we would follow up later based on the participant’s response. Lastly, our consent form and thank-you email post-debrief included information about farmer mental health resources.

The most important approach to preventing distress is to create a psychologically safe space where participants feel empowered to tell their story free of judgement. In the recruitment phase, the consent form, and the introduction to the group debrief, we explained that the project’s goal was to provide participants an opportunity to share their experiences with other women also raising children on farms, no matter how similar or different their experiences. Participants were told during the group debrief that there were no right or wrong ways of taking pictures or of talking about them and that they could skip questions they did not want to answer. The last key step to creating a psychologically safe space is maintaining confidentiality, as discussed above.

#### How Risk Unfolded During the Photovoice Activity

The photovoice activity proceeded as expected regarding the psychological risks. The pictures, particularly the ones responding to the prompt: “how does thinking about juggling children, farm work, taking care of the household, and off-farm work make you feel” led to a range of creative pictures. For example, one showed a women covered in manure with her thumb down; another, a very messy kitchen. In turn, these pictures generated rich discussions around quality of life and mental health. Meanwhile, participants frequently talked about being thankful for the space to discuss their lived realities raising children on a farm and for the understanding that they are not the only ones struggling. Several participants noted that while farmers’ mental health resources have developed in recent years, they have felt excluded because most target men and they were not aware of resources targeting the specific needs of farm women, including those with pre- and post-partum depression. Across group debriefs, participants played an active role in creating and maintaining a safe psychological space for one another. They made frequent comments about each other’s pictures, providing support, validation, and admiration. Several indicated that they would appreciate participating in informal group gatherings with other farm women because it helped them feel seen and understood. In other words, the group debrief did appear to adhere to some of the emancipatory nature of photovoice.

Across the fourteen photovoice debriefs, we only noticed one visibly distressed person, and we followed the protocol by sending her a private chat and sending a follow-up email right after the debrief ended. While a phone call might have been more effective to connect, we had not sought authorization from our REC to collect phone numbers.

### Social and Economic Risks to Researchers Associated with Veering From Disciplinary Norms

#### Source of Risk(s)

Though risk to researchers is not part of REC protocols in the United States, the photovoice activity could pose social and economic risks to the team. This is because the focus of the activity—on farm women’s lived realities and perspectives connected to raising children and on how childcare shapes their children’s exposure to risks on the farm—would likely lead participants to send pictures of children on the farm worksite including situations deemed dangerous by farm safety experts. As such, the photovoice activity would lead us to go against the established norms around visual representations in the agricultural health and safety field, as discussed above.

#### Addressing the Risk

Our approach to reducing social and economic risks to ourselves is part of ongoing discussions within our team and with colleagues. Receiving guidance from agricultural health and safety colleagues to provide educational material when seeing dangerous material gave us an indication that when communicating about the project, we needed to develop strategies to not only frame how we would show the pictures but also to explain the epistemological underpinnings of photovoice.

#### How Risk Unfolded During the Photovoice Activity

As noted above, we received a number of pictures that farm safety professionals would likely deem as unsafe. While for some of the participants, having the children around on the farm was seen as an important aspect of socialization, for more, the group debrief revealed that the situation depicted on the pictures was most often a result of lack of alternative childcare choices, which then led to rich discussions around their constant worries connected to safety and their strategies to keep the children safe. In the sharing of research findings (and in preparation for the public exhibit), we have done the following before showing pictures depicting dangerous situation. Without going too much into the details of photovoice as a methods, we explain that a basic principle of photovoice is: “We can’t fix what we can’t see.” We have also explained that the pictures and their captions represent what women choose to share to show their lived realities and perspectives and that some of these pictures may not be in line with farm safety recommendations. This strategy is informed by conversations with the project advisory board, which includes, among others, four farm women and two farm safety professionals. This strategy was approved by a lead agricultural health and safety scholar. The process of seeking buy-in through frequent conversations with agricultural health and safety colleagues takes time. Furthermore, it could be seen as taking power away from the women. Rather, our intent with seeking their buy-in is instead to create an open space to share the women’s stories.

## Lessons (Re)learned

Competing tensions between research methods, disciplinary social norms, and the REC regulatory framework can be frustrating and inhibit the development of participatory projects such as photovoice. The goal of this article was to leverage our experience developing a farm children safety photovoice project to provide practical guidance for future photovoice projects on agricultural health and safety topics. We conclude our article with three lessons we (re)learned.

First, photovoice led to the generation of new insights for the farm safety field while providing a space for engagement for women raising children on farms. In almost all group debriefs, women thanked us for having created this space for them to share with other farm women. However, it is perhaps the hardest and most time intensive REC protocol we have developed over the thirty years of combined research experience among the research team. In line with previous recommendations, our engagement with our RECs early and often was instrumental in learning about how the protocol would be reviewed and in collaboratively thinking through harder aspects of the protocol (Cross, Pickering, and Hickey 2015; McDonald and Capous-Desyllas 2021). We are, however, aware that not all RECs are able or willing to engage with researchers the way ours was. Engaging with others who have developed and implemented a photovoice project is valuable. Particularly useful to us was the ability to work from a previously approved REC consent form and a checklist used by an REC to review photovoice protocols. Neither document was publicly available; they were provided after we contacted researchers and RECs. One-on-one interactions should of course not be a substitute for engaging with the extensive peer-reviewed literature and practical guidelines. Among others, *The International Journal of Qualitative Methods*, Wang and Burris (1997), Wang and Redwood-Jones (2001), Cox et al. (2014), Sutton-Brown (2014), Jongeling et al. (2016), Hunger Free Colorado (n.d.) were all important resources.

Second, after reflecting on the deployment of the photovoice activity, we would make three changes to our protocol to be better prepared when working towards limiting psychological risks not only to the participants but also to us, the researchers. First, we would provide a list of mental health resources targeted to women in addition to those targeted to the general farm population. While we only noticed one participant visibly distressed, the topic of mental health challenges, including pre- and post-partum depression and perception that there is not enough support for women, was common across the group debriefs. Second, and closely connected to the first point, we would budget for a mental healthcare professional to be available as a first point of contact should our research participants want to debrief. Third, we would spend time thinking about how this project would impact our own well-being and the strategies we would use to alleviate the impact on our mental health. Creighton et al. (2018) and Alessi and Kahn (2022) provide insightful guidelines into trauma-informed qualitative research approaches. Hearing about the struggles faced by research participants, some of which we also experience in our own lives, was challenging for the team. While the research team had a thirty-minutes debrief after each photovoice debrief, in hindsight we failed to anticipate the impact of this project on our mental health. Self-care for researchers is increasingly being talked about in some fields (see, for example, Rager 2005 and Schulz et al. 2022). Admittedly, this is not a topic that is discussed in our areas of research but one that we plan on incorporating in protocol development moving forward.

Third, the group debriefs are an important time to realize the emancipatory power of photovoice for participants (besides the public photography exhibit which we do not discuss in this article). The COVID-19 restrictions meant that we conducted the photovoice activity online. While participants indicated that not having to travel eased their ability to participate, the online format felt more formal compared to previous experiences facilitating in-person group discussions with farm women. The informal conversations as we waited for everyone to join felt limited. It was also common for participants to only start engaging with the group once we formally started the debrief (e.g., waiting to turn cameras on, stepping away from the computer). At the end of the group debrief, participants did not have an opportunity to mingle with one another or even with the researchers. One way to foster a more informal debrief and/or to provide room for social interactions in a virtual environment would be to offer the use of virtual break out rooms and to build in social time, including time without the researchers in the Zoom room.

## Footnotes

1 Photovoice projects normally include a research and an outreach phase. For reasons we explain below, we decoupled the outreach from the research phase in our research ethics committee protocol. Our description and reflections of the methodological and ethical aspects in this paper are largely focused on the research phase.

2 We will use the more generic term throughout this paper; but note that institutional review board (IRB) is more commonly used in the United States.

3 U.S. Department of Health & Human Services regulations for the protection of human subjects in research. https://www.hhs.gov/ohrp/regulations-and-policy/regulations/45-cfr-46/index.html.

4 This statement is based on researchers’ interactions with farm safety colleagues about the visual representation of children in the worksite, including in the early stages of development of this photovoice project. Also see Childhood Agricultural Safety Network (2019) for an example of guidelines farm safety outreach professionals have developed for news media.

5 We thank special issue editor, Dr. Christopher Mayes, for suggesting ag-gag laws as a factor that could increase the difficulty of developing a photovoice project. While we had not thought about ag-gag laws in the development of our original protocol, they are important to consider given the negative consequences of these laws.

6 Minimal risk in the wording of U.S. regulations: “The probability and magnitude of harm or discomfort anticipated in the research are not greater than those ordinarily encountered in daily life or during the performance of routine physical or psychological examinations or tests” (45 CFR 46.102(j))
